# Up-Regulation of A1M/α_1_-Microglobulin in Skin by Heme and Reactive Oxygen Species Gives Protection from Oxidative Damage

**DOI:** 10.1371/journal.pone.0027505

**Published:** 2011-11-11

**Authors:** Magnus G. Olsson, Maria Allhorn, Jörgen Larsson, Martin Cederlund, Katarina Lundqvist, Artur Schmidtchen, Ole E. Sørensen, Matthias Mörgelin, Bo Åkerström

**Affiliations:** 1 Division of Infection Medicine, Department of Clinical Sciences, Lund University, Lund, Sweden; 2 Division of Dermatology and Venereology, Department of Clinical Sciences, Lund University, Lund, Sweden; Roswell Park Cancer Institute, United States of America

## Abstract

During bleeding the skin is subjected to oxidative insults from free heme and radicals, generated from extracellular hemoglobin. The lipocalin α_1_-microglobulin (A1M) was recently shown to have reductase properties, reducing heme-proteins and other substrates, and to scavenge heme and radicals. We investigated the expression and localization of A1M in skin and the possible role of A1M in the protection of skin tissue from damage induced by heme and reactive oxygen species. Skin explants, keratinocyte cultures and purified collagen I were exposed to heme, reactive oxygen species, and/or A1M and investigated by biochemical methods and electron microscopy. The results demonstrate that A1M is localized ubiquitously in the dermal and epidermal layers, and that the A1M-gene is expressed in keratinocytes and up-regulated after exposure to heme and reactive oxygen species. A1M inhibited the heme- and reactive oxygen species-induced ultrastructural damage, up-regulation of antioxidation and cell cycle regulatory genes, and protein carbonyl formation in skin and keratinocytes. Finally, A1M bound to purified collagen I (K_d_ = 0.96×10^−6^ M) and could inhibit and repair the destruction of collagen fibrils by heme and reactive oxygen species. The results suggest that A1M may have a physiological role in protection of skin cells and matrix against oxidative damage following bleeding.

## Introduction

The skin is continuously subjected to oxidative stress from both exogenous and endogenous sources. Reactive oxygen species (ROS) are generated from mitochondrial metabolism in dermal cells and enzymatic production by phagocytic and non-phagocytic NADPH-oxidases, but also from exposure of skin to ionizing radiation, UV-light irradiation, environmental toxins, chemotherapeutic compounds, etc. [1], [2], [3], [4]. ROS include hydrogen peroxide (H_2_O_2_) and the hydroxyl and superoxide radicals, which induce oxidative stress by oxidative reactions with cellular and extracellular molecular components. Normally, ROS and other oxidants are counteracted by antioxidants including the high-molecular weight enzymes superoxide dismutase (SOD), catalase, glutathione peroxidases (GPx) and the low-molecular weight non-enzymatic compounds glutathione (GSH), vitamin C and E [5], [6], [7], [8]. The molecular damage mechanisms of oxidative stress in skin are not fully known, but increased oxidative stress and insufficient antioxidant defenses are believed to play an important role in many skin diseases [9].

In conditions characterized by capillary leakage in the skin, such as during wounding, extra-vascular accumulation of red blood cells represents an oxidative threat because subsequent hemolysis leads to a massive release of free hemoglobin (Hb). Hb consists of four globin chains each carrying an iron-containing heme-group that is responsible for the oxygen-binding capacity of the molecule [10]. Free Hb is a strong pro-oxidant because it undergoes spontaneous auto-oxidation reactions generating ROS, free heme and free iron [11]. In addition, the latter two compounds are further sources of ROS and strong inducers of oxidative stress [12], [13].

Uncontrolled, excessive oxidative stress is believed to be a major factor during impaired wound repair [9]. We recently suggested a protective role of the antioxidant and heme- and radical scavenger α_1_-microglobulin (A1M) in chronic leg skin-wounds [14]. A1M is a ubiquitous low molecular weight (26 kDa) plasma and tissue protein [15], [16] mainly synthesized in liver [17], [18] but also, in less amounts, in peripheral organs such as blood cells, pancreas and kidney. From the liver cells, it is secreted to the blood stream where it is found in a free form (∼1 µM) and as high-molecular weight complexes with IgA, albumin and prothrombin (∼1 µM) [19], [20]. It is rapidly distributed to all tissues where it is transported from the blood vessels to the extravascular compartments [21]. A1M binds to free heme-groups [22], [23] and a heme-degrading truncated variant (t-A1M), lacking the C-terminal tetrapeptide LIPR, is formed in a reaction with free Hb [22]. A1M is a reductase [24], a multispecific scavenger of small organic radicals [25] and has antioxidant properties [26]. An increased synthesis in liver and blood cells is induced by cell-free Hb and ROS [27].

A1M is associated to both cells and matrix components in the epidermal and dermal layers of the skin [14], [28], [29]. In this work we have investigated the expression and localization of A1M in skin and keratinocytes, and the protective effects of A1M on *ex vivo* skin explants, keratinocyte cultures and purified collagen exposed to free heme and ROS.

## Results

### Localization and expression of A1M in skin and keratinocytes

**Figure 1:**
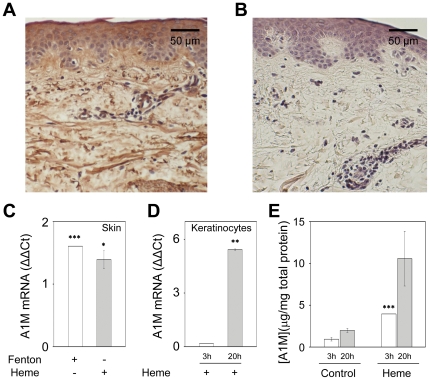
Histochemical distribution and expression of A1M. **A.** The sections (x10) were stained for A1M using monoclonal mouse anti-A1M BN11.10 (10 µg/mL) followed by peroxidase/diaminobenzidine tetrahydrochloride incubation as described in Materials and Methods. **B**. Control sections (x10) incubated without primary antibody. **C, D and E.** Human skin explants (1×10×2 mm) or primary keratinocytes were cultured in serum-free medium as described in Materials and Methods. **C.** Skin explants were incubated with 20 µM (NH_4_)Fe(SO_4_)_2_+200 µM ascorbate+40 µM H_2_O_2_ ( = Fenton; white bar) or 20 µM heme (grey) for a period of 20 hours. **D.** Keratinocytes were incubated with 20 µM heme for a period of 3 (white) or 20 (grey) hours. Total RNA was extracted from homogenized cells, cDNA was prepared using reverse transcription and expression of A1M was analyzed using real-time PCR as described in Materials and Methods. A1M threshold cycle values were normalized against G3DPH and ΔΔCt was calculated by normalizing against control samples which were incubated with buffer only for the same time-periods. Consequently, ΔΔCt-values of all controls are zero and correspond to the baseline. **E.** Keratinocytes were cultured in serum-free medium as described in Materials and Methods. The cells were then incubated with 20 µM heme for a period of 3 (white) or 20 (grey) hours. The cells were homogenized and the A1M concentration was determined using RIA as described in Materials and Methods. The total protein concentrations were determined with Bradford protein assay as described in Materials and Methods. Results are from triplicate experiments and are presented as mean ± SEM. Statistical comparison with control cultures was made using Students *t* test. * *P*<0.05 ** *P*<0.01, *** *P*<0.001.

A1M is ubiquitously localized in healthy skin as revealed by immunohistochemical staining of paraffin-embedded skin tissue sections (Fig 1A, B). Pericellular and cytosolic staining with anti-A1M was seen in the epidermal keratinocytes, slightly less intense in the deep layers. A global staining was seen in the dermis, and was especially pronounced in fibers, in the basement membranes around blood vessels, and in the epidermal-dermal junction.

Expression of the A1M gene in skin and in primary keratinocyte cultures obtained from skin was shown by real time PCR and radioimmunoassay (RIA) (Fig 1C, D and E, Table 1). The expression was significantly up-regulated after incubation of the skin explants or keratinocyte cultures with heme or hydroxyl radical-producing Fenton reaction-mixture for 20 hours. Furthermore, the synthesis of the A1M-protein was increased after incubating keratinocytes with heme for 3-20 hours (Fig 1E).

**Table 1:** Ct-values of A1M, HO-1 and p21 mRNA expression.

	A1M	HO-1	p21
**Skin**	*Normalized Cycle threshold (Ct) value*		
Control	29.54±0.42	30.90±0.19	33.20±0.00
20 µM heme	28.15±0.15^*1^	26.21±0.08^***1^	30.68±0.10^***1^
20 µM Fenton	27.93±0.00^***1^	28.47±0.23^**1^	32.29±0.01^***1^
20 µM heme+10 µM A1M	-	33.57±0.04^***2^	33.60±0.00^***2^
20 µM Fenton+10 µM A1M	-	31.14±0.03^***2^	33.80±0.30^***2^
**Keratinocytes**	*Normalized Cycle threshold (Ct) value*		
Control	34.43±0.27	25.01±0.04	-
20 µM heme	29.01±0.04^**1^	17.40±0.03^***1^	-
20 µM heme+10 µM A1M	-	19.27±0.03^***2^	-

**Figure 2:**
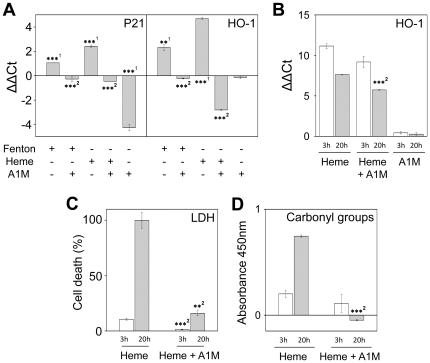
A1M inhibits heme- and ROS-induced oxidation in skin and keratinocytes. Human skin explants (1×10×2 mm) or primary keratinocytes were cultured in serum-free medium as described in Materials and Methods. **A.** Skin explants were incubated with 20 µM (NH_4_)Fe(SO_4_)_2_+200 µM ascorbate+40 µM H_2_O_2_ ( = Fenton), 20 µM heme with or without 10 µM A1M, or 10 µM A1M alone, for a period of 20 hours. **B.** Keratinocytes were incubated with 20 µM heme with or without 10 µM A1M, or 10 µM A1M alone, for a period of 3 (white) or 20 (grey) hours. Total RNA was extracted from homogenized cells; cDNA was prepared using reverse transcription and quantified by real-time PCR as described in Materials and Methods. p21 and HO-1 threshold cycle values were normalized against G3DPH and ΔΔCt were calculated by normalizing against non-exposed samples. Consequently, ΔΔCt-values of all controls are zero and correspond to the baseline. **C and D.** Keratinocytes were cultured in serum-free medium as described in Materials and Methods. The cells were then incubated with 20 µM heme with or without 10 µM A1M for a period of 3 (white) or 20 (grey) hours. **C.** The amount of LDH present in the culture medium was measured using CytoTox 96® Non-Radioactive Cytotoxicity Assay as described in Materials and Methods. **D.** The cells were harvested and the protein carbonyl group concentrations were measured by ELISA as described in Materials and Methods. Results are from triplicate experiments and are presented as mean ± SEM. Statistical comparison between groups was made using Students *t* test. * *P*<0.05 ** *P*<0.01, *** *P*<0.001. ^1^ Statistical comparison vs. control, ^2^ statistical comparison with heme or Fenton treatment.

cDNA was prepared using reverse transcription and quantified by real-time PCR as described in Materials and Methods. A1M, HO-1 and p21 threshold cycle values were normalized against G3DPH. Results are from triplicate experiments and are presented as mean ± SEM. Statistical comparison between groups was made using Students *t* test. * *P*<0.05, ** *P*<0.01, *** *P*<0.001.^1^ Statistical comparison vs. control, ^2^ statistical comparison vs heme or Fenton-reaction. The Ct-values were also used to calculate ΔΔCt-values and these are shown in Figures 1 and 2.

### A1M inhibits heme- and ROS-induced oxidative damage in skin and keratinocytes

**Figure 3:**
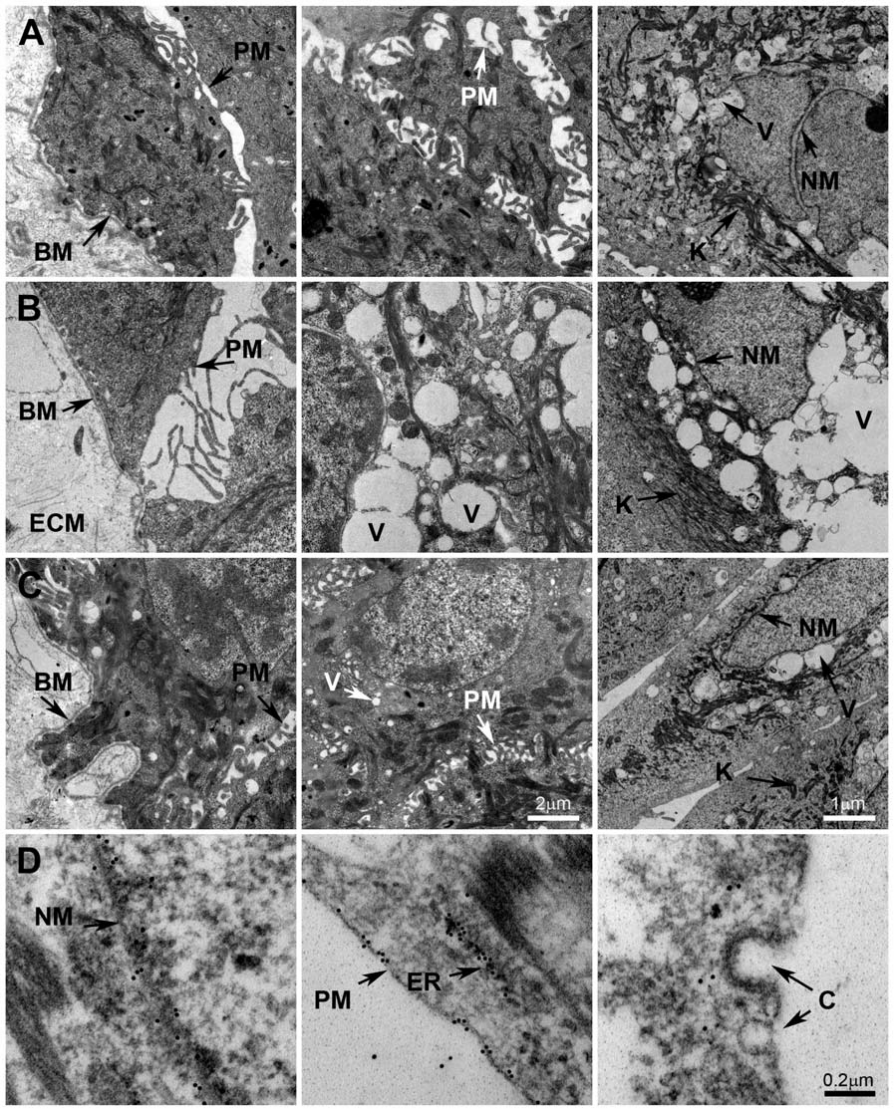
Human normal skin (Left and middle panel) was dissected into 1×10×2 mm pieces and placed in Keratinocyte SFM culture medium. Human primary keratinocytes were cultured in Keratinocyte SFM medium (**Right** panel). Skin and keratinocytes were then incubated for 20 hours at RT with buffer only (**A**), with 20 µM heme (**B**) or with 20 µM heme and 10 µM A1M (**C, D**). Immunolabeling of thin sections with gold-labeled anti-A1M were performed (**D**). The samples were prepared as described in Materials and Methods and observed in a Jeol JEM 1230 electron microscope, operated at 80 kV accelerating voltage. Images were recorded with a Gatan Multiscan 791 charge-coupled device camera. BM =  basement membrane, PM = plasma membrane, NM =  nucleus membrane, V =  vacuoles, ECM =  extracellular matrix, K =  keratin, ER =  endoplasmatic reticulum, C =  caveoli. Scale bar in **C** (**middle**) indicate 2 µm (is applicable for Figure **A**, **B** and **C**, **left** and **middle**), in **C** (**right**) indicate 1 µm (is applicable for **A**, **B** and **C**, **right**) and **D** (**right**) indicate 0.2 µm (is applicable for **D**, left, middle and right).

Skin and keratinocytes were incubated *in vitro* with heme and the Fenton reaction-mix, in the presence or absence of A1M, and the response was analyzed using molecular biological and biochemical parameters (Fig 2) and transmission electron microscopy (EM) (Fig 3).

In skin, both heme (20 µM) and the Fenton reaction-mix (20 µM), incubated with the explants for 20 hours, induced a significantly increased expression of the cell cycle regulatory gene p21, and heme oxygenase-1 (HO-1), a heme-degrading enzyme shown to be up-regulated by ROS and oxidants [30](Fig 2A, Table 1). The increased expression of these two genes was inhibited by the addition of 10 µM A1M, down to below the levels of un-stimulated skin. In keratinocytes, 20 µM heme, incubated for 3 or 20 hours, induced a significantly increased expression of the HO-1 gene, and this was also significantly lower in the presence of 10 µM A1M (Fig 2B). Addition of 10 µM A1M alone significantly down-regulated the expression of p21 in skin explants (Fig 2A) but had no effect on the expression of HO-1-gene in skin or keratinocytes (Fig 2A and B), indicating different regulation mechanisms of these two genes. Finally, a significantly increased cell-death (Fig 2C) and formation of the oxidative marker protein carbonyl groups (Fig 2D) were observed. These responses were significantly lower in the presence of 10 µM A1M (Fig 2C-D). Indeed, after 20 hours, the cell-death of the keratinocytes was almost 100% in the presence of heme, but suppressed down to 15% by addition of A1M (Fig 2C).

To further analyze the protection by A1M, EM was performed on skin explants (Fig 3A–C, left and middle columns) and cultured keratinocytes (Fig 3A–C, right column). Extensive destructive effects were seen by heme both in skin and keratinocytes, (Fig 3B), i.e. vast formation of vacuoles, formation of intercellular gaps, ruffling and rupture of plasma membrane and structural des-organization of keratin fibers. These effects were counteracted by the addition of A1M (Fig 3C). Furthermore, heme incubation resulted in a loss of dense structures in the extracellular matrix architecture (Fig 3B, left). This effect was reversed by addition of A1M (Fig 3C, left). Incubation with the Fenton reaction-mix induced similar effects on skin and keratinocytes, and these were also counteracted by A1M (not shown). The results suggest that A1M protects and preserves cellular structures otherwise damaged and disintegrated by heme and ROS.

EM of tissue specimens, containing exogenously added A1M and incubated with gold-labeled anti-A1M, showed a localization of A1M to the plasma membrane, ER and nuclear membrane (Fig 3D). The anti-A1M was not associated with caveoli (Fig 3D, right) suggesting a non-endocytotic uptake of the protein by the keratinocytes.

### A1M inhibits and reverses heme- and ROS-induced oxidation of collagen I *in vitro*

**Figure 4:**
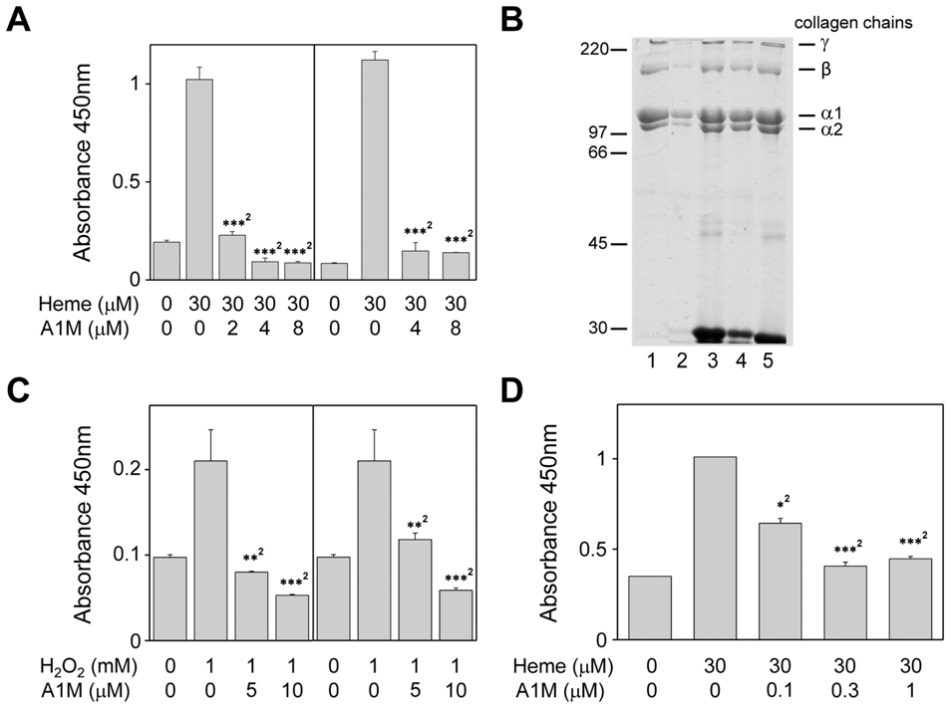
A1M inhibits and repairs heme- and ROS-induced oxidation of collagen I *in vitro*. A (Left). Collagen I, coated to microtiter plates, was incubated with 30 µM heme alone or in the presence of A1M (2, 4, or 8 µM). **(Right)** Collagen, coated to microtiter plates, was pre-incubated with 4 or 8 µM A1M and washed before the addition of 30 µM heme. After 4 hours incubation the protein carbonyl group concentration was measured using ELISA as described in Materials and Methods. **B.** Collagen (5 µM) was incubated with PBS (lane 1), 160 µM H_2_O_2_ (lane 2), 160 µM H_2_O_2_+10 µM A1M (lane 3), 160 µM H_2_O_2_+3 µM A1M (lane 4) or only 10 µM A1M (lane 5). After 2 hours at 25°C, the samples were separated on 8% SDS-PAGE and stained with Coomassie Brilliant Blue R-250. **C (Left).** Collagen, coated to microtiter plates, was incubated for 4 hours with 1 mM H_2_O_2_ alone, or a mixture containing 1 mM H_2_O_2_ plus 5 µM or 10 µM A1M. **(Right)** Collagen, coated to microtiter plates, was pre-incubated with A1M (5 or 10 µM) and washed before the addition of 1 mM H_2_O_2_. The protein carbonyl group concentration was measured using ELISA as described in Materials and Methods. **D.** Collagen, coated to microtiter plates, was oxidized by incubation with 30 µM heme for 17 h. After washing, 0.1, 0.3 or 1 µM A1M was added, and incubated for 2 hours. The protein carbonyl group concentration was measured by ELISA as described in Materials and Methods. Statistical comparison between groups was made using Students *t* test. * *P*<0.05 ** *P*<0.01, *** *P*<0.001. ^2^ statistical comparison with heme or Fenton treatment.

The results obtained by immunohistochemistry and EM suggest a localization of A1M to collagen fibers and a protective effect of the protein in the extracellular matrix. Therefore, the effects of heme and ROS and the protective properties of A1M on purified collagen I were investigated. Heme induced carbonyl group formation on collagen monomers immobilized to microtiter plates and this was completely inhibited by addition of A1M (Fig 4A, left) in a dose-dependent manner. A1M was also pre-incubated with collagen and non-bound A1M was removed by washing before addition of heme. As shown in Fig 4A (right), the carbonyl formation on collagen was inhibited by A1M suggesting that the bound A1M was sufficient to achieve the inhibition.

As shown in Fig 4B the exposure of collagen to 0.16 mM H_2_O_2_ resulted in disappearance of the collagen bands, suggesting an extensive degradation of the collagen. The degradation was inhibited by addition of 3 or 10 µM A1M but not by the lipocalin α_1_-acid glycoprotein (not shown). Exposure of collagen to 1 mM H_2_O_2_ also induced formation of carbonyl groups in the microtiter plate assay, and this was inhibited by addition of 5 or 10 µM A1M in a dose-dependent manner (Fig 4C, left). Pre-incubation of collagen with A1M followed by washing before addition of H_2_O_2_, also resulted in a dose-dependent inhibition (Fig 4C, right) suggesting that A1M bound to collagen was sufficient to achieve the inhibition.

In most experiments the ELISA absorbance values in the presence of A1M were even lower than in untreated collagen (Fig 4A–C). This suggests that A1M may be able to remove pre-formed oxidation products. To investigate this, collagen was oxidized on microtiter plates with 50 µM heme and washed before A1M was added (Fig 4D). A significant decrease of carbonyl groups was seen after addition of 0.1 µM A1M and was even more evident after addition of 0.3 and 1 µM A1M. The control substances, ovalbumin and ascorbate, the latter an antioxidant and reducing agent, did not have any significant effects at these concentrations (not shown).

**Figure 5:**
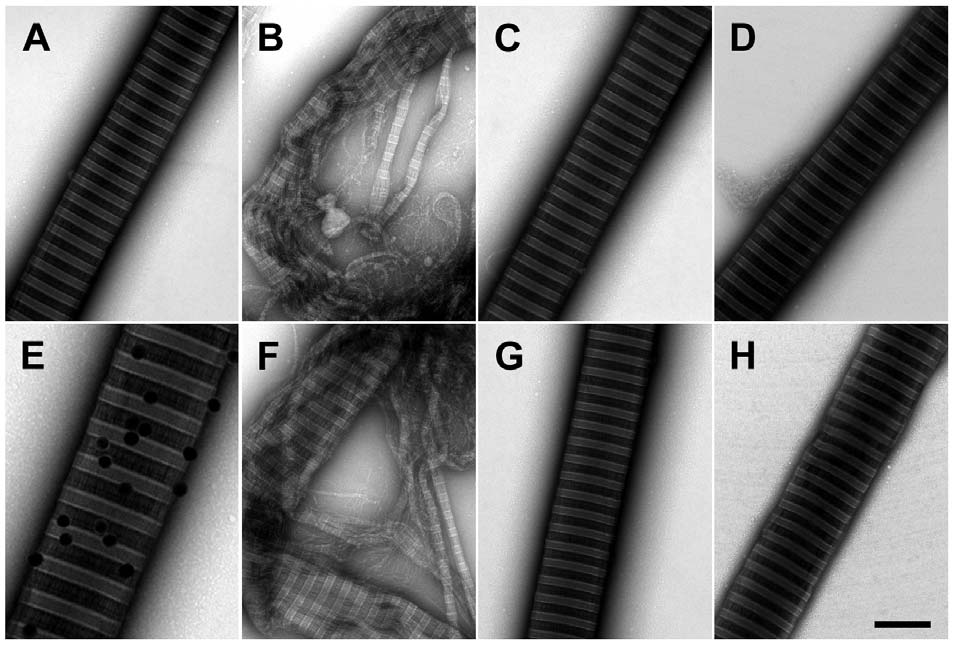
A1M inhibits destruction of collagen fibrils. Collagen was incubated with buffer for 24 hours at RT to allow fibrillation. Fibrils were then incubated for a second 24-hour period at RT with heme (**B**, 20 µM), Fenton-reaction mixture (**F**, 100 µM Fe^3+^, 1.0 mM ascorbate, 200 µM H_2_O_2_) or buffer only (**A**), either with or without A1M (**C** and G,10 µM). Fibrils incubated without A1M during the second 24 hour period were then incubated for a third 24 hour period with buffer or 10 µM A1M (**D** and **H**). The samples were then adsorbed for 1 min onto carbon-coated grids. To study binding of A1M to collagen (**E**), collagen was allowed to form fibrils by incubation with buffer and then incubated with gold-labeled A1M for 1 hour at RT. The samples were analyzed in a Jeol 1200 EX electron microscope operated at 60 kV accelerating voltage. The scale bar indicates 50 nm in **A–D** and **F-H** and100 nm in **E**.

Next, the effects of heme, the Fenton reaction-mix, and A1M on collagen fibrils, formed *in vitro* from purified collagen I-monomers, were examined by EM (Fig 5). Disruption of the fibrils (Fig 5A) was seen after 24 hours incubation with 20 µM heme (Fig 5B) or Fenton reaction-mix (100 µM Fe^2+^, Fig 5F). Addition of 10 µM A1M to the incubations with heme (Fig 5C) or the Fenton reaction-mix (Fig 5G), prevented the destruction. As seen in Fig 5D and 5H, incubation with 10 µM A1M after the heme- or Fenton incubation reversed the destructive effects on the fibril morphology.

### Binding of A1M to collagen fibrils

**Figure 6:**
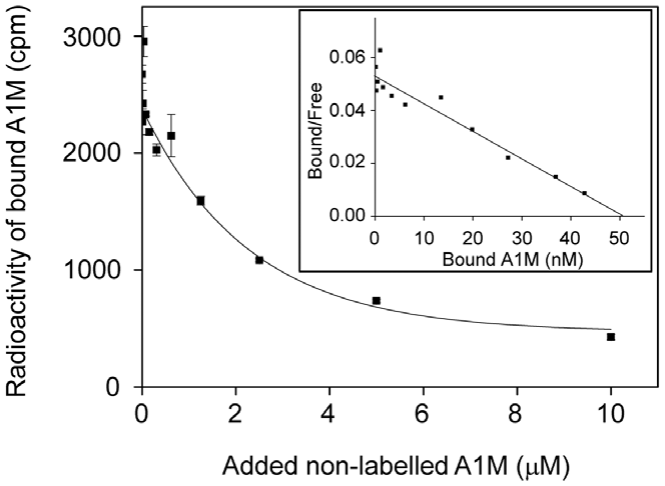
Binding of A1M to collagen I. Purified collagen I-monomers were coated to microtiter plates and incubated with ^125^I-labelled A1M in the presence of increasing amounts of non-labeled A1M. After washing, the radioactivity bound to the microtiter plate walls was plotted against the total concentration of A1M. The binding displacement-curve is shown with the corresponding Scatchard plot (insert). The strength of the binding was estimated using Scatchard analysis, giving a K_d_ of 0.96×10^−6^ M. Each point represents the mean ± SEM of three determinations.

A1M bound *in vitro* to the collagen fibrils, as revealed by EM using gold-labeled anti-A1M antibodies (Fig 5E). A specific binding of ^125^I-labelled A1M was also demonstrated and quantified after coating of microtiter plates with collagen I monomers (Fig 6). Using Scatchard analysis, the binding strength was estimated to 0.96×10^−6^ M^−1^.

## Discussion

Immunohistochemistry revealed a broad distribution of A1M in the epidermal and dermal layers of the skin. The protein has previously been found in normal skin, but only at specific locations: epidermis, the epidermal-dermal junction [28], at occasional dermal fibrous structures [29] and in the basement membranes around blood-vessels in skin from chronic leg ulcers [22]. The findings here, however, are the first evidence of an extensive presence of A1M in both epidermal and dermal layers of normal skin (Fig 1). Moreover, a constitutive expression of A1M was demonstrated in epidermal keratinocytes. Cytoplasmic staining of cells in the epidermis by anti-A1M was apparent in the immunohistochemical sections (Fig 1). The expression of the A1M-gene and production of the A1M-protein in cultured keratinocytes could be confirmed by real-time PCR and RIA (Table 1, Fig 1). The results indicate a similar level of A1M-synthesis as previously reported in cells of myeloid and erythroid origin [27] but a lower level than in the liver and cells of hepatic origin [18], [27], [31]. These results thus corroborate the view of A1M as synthesized constitutively at a high level in the liver and at a lower level in most other organs.

The expression of A1M in keratinocytes was up-regulated by exposure to free heme or iron-generated hydroxyl radicals (Fig 1). We therefore hypothesized that the increased production of A1M would lead to protection of the keratinocyte cultures and skin against oxidative damage caused by heme and ROS. The results reported here support this hypothesis. Thus, addition of A1M inhibited the heme- and ROS-induced cell-death, formation of carbonyl groups and up-regulation of cell cycle arrest (p21) and antioxidation (HO-1) genes in skin and keratinocytes. A similar up-regulation of A1M and protective effects against Hb-, heme- and ROS-induced oxidative damage was reported previously in hepatoma and blood cell-lines [26], [27].

EM analysis of skin tissue and keratinocyte cultures after incubation with heme, revealed signs of apoptosis (vacuole formation), plasma membrane rupture, destruction of the structure of the endoplasmatic reticulum and nuclear membrane, and a reduction of the keratin fibers in number and thickness. Similar effects were seen when exposing skin and keratinocytes to the Fenton reaction (not shown). The ultrastructural tissue damage seen here resemble the damage of trophoblasts described after *in vitro* perfusion of placentas with Hb [32], suggesting similar mechanisms for the oxidative destruction of skin and placenta ultrastructure by Hb, free heme or iron. In the same placenta perfusion study [32], A1M inhibited the Hb-induced cell- and matrix damage as seen by EM analysis, and similar results were obtained in this study, i.e. A1M inhibited the heme- and iron-induced morphological destruction of the skin and keratinocytes. Furthermore, A1M was found to be associated with most of the organelles through-out the cell, i.e. localized to the plasma membrane, endoplasmatic reticulum, Golgi, mitochondria and nuclear membrane by immunostaining of EM-sections with anti-A1M (Fig 3). The ubiquitous intracellular localization may contribute to the efficient cytosolic protective effects of the protein.

The immunohistochemical analysis also suggests a localization of A1M to collagen fibers in the extracellular matrix (Fig 1), supporting previous reports on skin and placenta [28]. Here, we demonstrate a direct binding between purified A1M and collagen I fibrils, using EM and binding assays, with an affinity in the physiological range (Figs 5E and 6). The results also demonstrated protective effects of A1M against oxidative damage of collagen fibers. The carbonyl group formation and fibrillar destruction induced by free heme and iron were inhibited by A1M. The EM-analysis also showed that A1M could protect extracellular matrix structures *ex vivo*, i.e. addition of A1M to skin explants exposed to heme and iron reversed the disintegration of extracellular matrix (Fig 3A-C, left panels). These results agree well with the previous report [32] that perfusion of placentas with Hb induced a dramatic loss of striated fibrils in the extracellular matrix, which was counteracted by A1M. Interestingly, perfusion with A1M also induced an up-regulation of collagen-, elastin-, and other genes of extracellular matrix components. Thus, taken together, the localization of A1M to matrix fibers, binding to collagen fibrils, inhibition of oxidative damage and up-regulation of collagen genes, suggest an important role of A1M in collagen homeostasis.

A1M has been shown to have a reductase activity [24], [25], as well as heme- and radical-binding properties [22], [23], [25] but the precise molecular mechanisms of its protective activities are not yet known and needs to be investigated in more detail. A unifying model of the protection mechanism of A1M may be that the molecule acts as a “radical sink”. According to this model, which was proposed i detail previously [25], each A1M-molecule can eliminate at least 8-9 radicals by a semi-catalytic covalent trapping mechanism that involves the reducing capacity of the Cys34 side-chain and intramolecular electron-transfer between several Tyr- and Lys-side chains and Cys34. These side-chains are thus oxidized to a one-electron radical state and can react with ROS and radicals in the near environment by covalent binding. The net result is the reduction of 5-6 radicals plus covalent binding of at least 3 other radicals to the A1M-molecule. The covalent trapping continues until the A1M-molecule becomes saturated and is cleared from the tissues by the blood and subsequent glomerular filtration in the kidneys. It can thus be speculated that the ROS-species generated both by the heme-group and by the Fenton-reaction are eliminated by this mechanism. Thus, in the case of the heme-group, it may not be the binding of heme-groups *per se* that inhibits the oxidative injuries induced by heme, but rather the scavenging of downstream ROS-species generated by free heme. This would explain the catalytic action of A1M *visavi* both heme- and Fenton-induced oxidative tissue damage, i.e. the higher than 1:1 molar capacity of A1M. We have shown previously that a mutated form of A1M, lacking the active group of Cys34, is not capable of reductase or radical scavenging activity [24], [25]. According to the protection model discussed here, this mutated form should not be able to exert any protection against heme- and Fenton-induced cell and tissue damage, even though it may still have the heme-binding capacity. We are presently investigating this hypothesis.

The results indicate that the oxidative modifications on collagen, measured as carbonyl groups, were reduced by incubation with A1M. Thus, A1M could not only inhibit the formation of oxidative modifications but also remove pre-formed carbonyl groups (Fig 4). This indicates a repair effect of the protein which also may be an effect of its reductase activity. Moreover, the results of the EM analysis (Fig 5) show that heme and iron destroyed the collagen fibrils. Adding A1M reversed the reaction, i.e. the fibrils were regenerated. Hypothetically, oxidative modifications of the monomers, induced by heme and Fenton reaction, may induce a shift of the equilibrium between monomers and fibrils towards the monomers by preventing their interaction, whereas A1M, by removing/reducing the oxidative modifications, thus may promote a shift of the equilibrium back towards fibril formation.

Many pathological conditions of the skin are associated with an increased oxidative insult to cells and tissue components. As discussed above, extravasation of red blood cells and subsequent hemolysis result in high levels of Hb, heme, iron and free radicals in chronic leg ulcers. Furthermore, microbial infections and many other inflammatory conditions, such as atopic dermatitis and psoriasis are associated with necrotic cell-death and release of free radical-generating cell-components such as mitochondria and cytochromes. The protective mechanisms against these destructive processes are not yet fully understood. The results presented in this paper suggest that the plasma and tissue protein A1M may have an important role as an anti-oxidant, protecting both cells and the extracellular matrix of skin during health and disease.

## Materials and Methods

### Reagents and proteins

Cell culture media and supplements were purchased from Invitrogen (Paisley, Scotland, UK). Hemin (Ferriprotoporphyrin IX chloride) was purchased from Porphyrin Products, Inc. (Logan, UT, USA) and a 10 mM stock solution prepared by dissolving in dimethyl sulphoxide (Sigma-Aldrich, St-Louis, MO, USA). “Heme” and “hemin” are sometimes used to designate free protoporphyrin IX with a bound Fe^2+^ or Fe^3+^ atom, respectively; in this article, “heme” is used regardless of the iron oxidation state. Hydrogen peroxide was bought from Acros Organics (Geel, Belgium). Ammonium iron (III) sulfate dodecahydrate was from Merck (Darmstadt, Germany). A mixture of Fe^3+^ (10–100 µM), ascorbate (0.1–1.0 mM) and hydrogen peroxide (20–200 µM) was used to generate hydroxyl radicals by the Fenton-reaction. In this manuscript, the concentration of the Fenton reaction-mix will be referred to as the Fe^3+^ concentration. Collagen S (soluble, type I, from calf skin, 3 mg/mL) was bought from Roche (Mannheim, Germany). CytoTox96® Non-Radioactive Cytotoxicity Assay was from Promega (Madison, WI, USA). 2,4-dinitrophenylhydrazine (DNP-hydrazine) and o-phenylenediamine were from Sigma-Aldrich). Anti-DNP-keyhole limpet hemocyanin (KLH) was from Invitrogen. Swine anti-rabbit IgG-horseradish peroxidase (HRP) was purchased from Dako A/S (Glostrup, Denmark). Recombinant human A1M, containing an N-terminal His-tag, was expressed in *E. coli* and purified and refolded as described [33] with the addition of an ion-exchange chromatography purification step. This was performed by applying the A1M to a column of DEAE-Sephadex A-50 (GE Healthcare, Uppsala, Sweden) equilibrated with the starting buffer (20 mM Tris-HCl, pH 8.0). A1M was eluted at a flow rate of 1 mL/min using a linear pH gradient consisting of 250 mL starting buffer in the first chamber and 250 mL elution buffer (20 mM Tris-HCl, 0.2 M NaCl, pH 8.0) in the second. Absorbance at 280 nm of eluted fractions was read and fractions containing pure A1M were pooled and concentrated. Goat antibodies were prepared against human urinary A1M using the procedure described [34]. Monoclonal mouse anti-A1M (BN11.10) was prepared and characterized as described [35], [36].

### Cell culture

Human primary keratinocytes (Cambrex Biologics, Karlskoga, Sweden) were cultured in Keratinocyte SFM medium (Invitrogen, Paisley, Scotland, UK) containing 100 µg/mL antibiotics and 0.25 µg/mL antimycotics (amphotericin B and Fungizone®). The cells were incubated at 37°C in 5% CO_2_. 20 µM heme and/or A1M (10 µM) were added to the cells as indicated in the figure legends. Culture medium was collected and cells were harvested in homogenizing buffer (containing 50 mM Tris-HCl, pH 8.0; 2 mM EDTA; 1% NP40; 1 µg/µL pepstatin, 5 µg/µL antipain; 10 µg/µL leupeptin) after 3 and 20 hours and analyzed as described below.

### Skin

Normal human skin obtained from reduction surgery was dissected into 1×10×2 mm pieces and two pieces were placed in each well of a 6-well culture dish containing Keratinocyte SFM culture medium (containing 100 µg/mL antibiotics). Heme (20 µM), Fenton reaction-mix (20 µM Fe^3+^, 0.5 mM ascorbate, 0.1 mM H_2_O_2_) and/or A1M (10 µM) were added to the skin pieces (as indicated in the figure legends), cultured for 5 hours at 37°C in 5% CO_2_ and analyzed using techniques described below. The skin specimens were surplus tissue from dermatological surgery, and obtained following written consent using protocols approved by the ethical committee at Lund-Malmö University Hospital (LU 762-02).

### Cell viability assay

The concentration of lactate dehydrogenase (LDH) in culture media was used to estimate the degree of cytolysis induced by heme. LDH was measured using CytoTox 96® Non-Radioactive Cytotoxicity Assay from Promega. The analysis was performed according to the instructions from the manufacturer. Briefly, at the end-point of all incubations culture medium was harvested and 50 µL was transferred to a 96-well microtiter plate. Fifty microliters reconstituted substrate mix were added to each well and the plate was incubated at room temperature (RT), protected from light. After 30 minutes 50 µL stop solution were added to each well and absorbance was read at 490 nm using a Wallac 1420 Multilabel Counter (Perkin Elmer Life Sciences, Waltham, MA, USA). A1M alone had no significant effect on the absorbance at 490 nm of the reagents.

### RNA isolation and real-time PCR

Total RNA was isolated from skin and keratinocytes using the acid guanidinium phenol chloroform method supplied by QIAGEN Sciences (Germantown, MD, USA). The OD ratio (optical density at 260 nm/280 nm) of RNA was always greater than 1.8. Reverse transcription was performed on 3 µg total RNA at 42°C for 60 min in the presence of 0.5 µg oligo(dT)_18_ primer, 200 U reverse transcriptase and 20 U RiboLock^TM^ Ribonuclease inhibitor in reaction buffer (RevertAid^TM^ H Minus First Strand cDNA Synthesis Kit, Fermentas GMBH, St. Leon-Rot, Germany). Real-time PCR was then used to quantify the p21, A1M and (HO-1) mRNA. Raw data were obtained as cycle threshold values (Ct-values) and are shown in Table 1 after normalization to the Ct-values of human glyceraldehyde-3-phosphate dehydrogenase (G3DPH). The ΔΔCt-values shown in the Figures were then calculated by normalizing against control samples from cells incubated with buffer only for the same time-periods. A lower Ct-value corresponds to an increased mRNA-level and is therefore depicted as an increased ΔΔCt-value, and *vice versa*. The ΔΔCt-values of the controls correspond to the baseline and are not included in the Figures. Primers were designed accordingly: p21 forward primer 5′-TGGACCTGTCACTGTCTTGT-3′, reverse primer 5′-TCCTGTGGGCGGATTAG-3′; A1M forward primer 5′-CACTCGTTGGCGGAAAGG-3′, reverse primer 5′-ACTCATCATAGTTGGTGTGGAC-3′; HO-1 forward primer 5′-CAACAAAGTGCAAGA TTCTG-3′, reverse primer 5′-AAAGCCCTACAGCAACTG-3′; G3DPH forward primer 5′-TGGTATCGTGGAAGGACTC-3′, reverse primer 5′-AGTAGAGGCAGGGATGATG-3′. The expression was analyzed using iQ SYBR Green Supermix (Bio-Rad, Hercules, CA, USA). Amplification was performed at 55°C for 40 cycles in iCycler Thermal Cycler (Bio-Rad) and data analyzed using iCycler iQ Optical System Software.

### Radioimmunoassay and total protein analysis

Radiolabelling of A1M with ^125^I was done using the chloramine T method [37]. Protein-bound iodine was separated from free iodide by gel-chromatography on a Sephadex G-25 column (PD10, GE Healthcare, Buckinghamshire, UK). A specific activity of around 0.1–0.2 MBq/µg protein was obtained. RIA was performed as described [38]. Briefly, goat antiserum against human A1M (0.2 mL, dil. 1:6000) was mixed with ^125^I-labelled A1M (0.1 mL, approximately 0.05 pg/mL) and samples, or standard A1M concentrations (0.2 mL). The dilutions were done in 0.1 M sodium phosphate, pH 7.4+0.1% BSA (RIA-buffer). After incubating overnight at RT, antibody-bound antigen was precipitated by adding 0.3 mL bovine serum and 1.6 mL 15% polyethylene glycol in the RIA-buffer, centrifuged at 2500 xg for 40 min, and the ^125^I activity of the pellets was measured in a Wallac Wizard 1470 gamma counter (Perkin Elmer Life Sciences). Total protein in cell-homogenates was determined by Bradford protein assay. This assay was performed by adding one mL of Bradford reagent (containing 4.2 µM Coomassie Brilliant Blue G (Sigma-Aldrich, St. Louis, MO, USA), 5% (v/v) EtOH, 6% (v/v) H_3_PO_4_ dissolved in H_2_O) to 100 µL of each sample, and incubating at room temperature for 5 min before determination of absorbance at 595 nm using a UV spectrophotometer (Beckman DU640; Beckman Instruments, Palo Alto, CA, USA). Albumin was used as a standard and plotting the absorbance at 595 nm versus protein concentration generated a standard curve.

### Determination of protein carbonyl groups in purified collagen and keratinocyte medium

Purified collagen S was coated overnight at 4°C to microtiter plates (Nunc Maxitorp, WVR International, Sweden) diluted to 5 µg/ml in 20 mM Tris-HCl, 140 mM NaCl, pH 7.6, washed five times with PBS+0.1% Tween-20 and incubated with 30 µM heme or 1 mM H_2_O_2_, together with A1M at different concentrations in PBS +0.1% Tween-20. Alternatively, the collagen-coated wells were first incubated with A1M at different concentrations in PBS +0.1% Tween-20, washed and then oxidized with 30 µM heme or 1 mM H_2_O_2_. Formation of protein carbonyl groups in the collagen was then quantified as described [39]. Briefly, 25 µL samples were mixed with 75 µL 10 mM DNP-hydrazine (solved in 6 M Guanidine hydrochloride, 0.5 M sodium phosphate, pH 3.0) for 45 minutes at RT. The DNP-hydrazine derivatized samples were then diluted with PBS and coated on a 96-well microtiter plate for 2 hours at RT. After rinsing, the plate was incubated with rabbit anti-DNP-KLH (diluted 1:2000 in PBS, 0.1% BSA, 0.25% Tween 20) for 2 hours at RT, followed by rinsing and incubation with swine anti-rabbit IgG-HRP (diluted 1:2000 in the same buffer) for 1 hour at RT. Finally, the plate was incubated with substrate solution (1 tablet o-phenylenediamine dissolved in 60 mM Tris-HCl, pH 8.5, 50 mM Na_2_HPO_4_, H_2_O_2_) and absorbance was read at 450 nm, using a Wallac 1420 Multilabel Counter, at the onset of the reaction until a peak absorbance was obtained. Protein carbonyl groups in keratinocyte medium was determined as described [40] by first DNP-derivatizing the medium samples, and then coating to microtiter plates and assay with anti-DNP-KLH using the conditions described above.

### Transmission electron microscopy (TEM)

For negative staining collagen S (5 µg/mL) was incubated with buffer (50 mM Tris-HCl, 0.15 M NaCl, pH 7.6) for 24 hours at RT to allow fibrillation. Fibrils were then incubated for a second 24 hour-period at RT with a Fenton-reaction mixture (100 µM Fe^3+^, 1.0 mM ascorbate, 200 µM H_2_O_2_), heme (20 µM) or buffer only, either with or without A1M (10 µM). Fibrils incubated without A1M during the second 24 hour-period were then incubated for a third 24 hour-period with buffer or 10 µM A1M. The samples were then adsorbed for 1 min onto carbon-coated grids, briefly washed with water, and stained with 0.75% (w/w) uranyl formate. The grids had been rendered hydrophilic by glow discharge at low pressure in air beforehand. To study binding of A1M to collagen, 5 µg/mL collagen S was allowed to form fibrils by incubation with buffer and then incubated with gold-labeled A1M (4-5 nm) for 1 hour at RT. A1M was labeled with colloidal gold as previously described (40).

For ultrathin sectioning, purified keratinocytes and skin sections were fixed for 1 hour at RT and then overnight at 4°C in 2.5% glutaraldehyde in 0.15 M sodium cacodylate, pH 7.4 (cacodylate buffer). Samples were then washed with cacodylate buffer and post-fixed for 1 hour at RT in 1% osmium tetroxide in cacodylate buffer, dehydrated in a graded series of ethanol, and then embedded in Epon 812 (SPI Supplies, West Chester, PA, USA) using acetone as an intermediate solvent. Specimens were sectioned with a diamond knife into 50–70 nm-thick ultrathin sections on an LKB ultramicrotome. The ultrathin sections were stained with uranyl acetate and lead citrate. Specimens were observed in a JEOL JEM 1230 electron microscope operated at 80 kV accelerating voltage. Images were recorded with a Gatan Multiscan 791 CCD camera. Keratinocytes (about 1 million cells) were pelleted by centrifugation and subsequently fixed and sectioned. Tissue samples were treated in a similar way, except that the centrifugation step was omitted. Immunolabeling of thin sections with gold-labeled anti-A1M were performed as described by Roth [41] with the modification that Aurion-BSA (Aurion, Wageningen, The Netherlands) was used as a blocking agent. Samples were finally stained with uranyl acetate and lead citrate and observed in a Jeol JEM 1230 electron microscope, operated at 80 kV accelerating voltage. Images were recorded with a Gatan Multiscan 791 charge-coupled device camera.

### Collagen degradation analysis

Collagen, 5 µM, was incubated with H_2_O_2_ (0.15–1 mM), A1M (0–10 µM), or both, in 50 mM Tris-HCl, 0.15 M NaCl, pH 7.6. After incubation for 1.5 hour at room temperature (RT), the samples were separated by SDS-PAGE as described previously [42].

### Immunohistochemistry

Immunohistochemistry sections were deparaffinized by routine procedures and endogenous peroxidase activity was blocked with 3% H_2_O_2_ in methanol for 10 minutes. After rinsing in distilled water, followed by PBS, the slides were subsequently permeabilized with Tween 20 (0.05%) in PBS. Thereafter, sections were rinsed in PBS. After pre-incubation with normal goat serum (1%) for 30 min at RT, incubation was performed with primary antibody (mouse monoclonal anti-A1M BN11.10) diluted to 10 µg/mL in PBS (containing 0.05% Tween 20, 0.2% BSA) overnight at 41°C. Control sections were incubated in the same PBS solution without antibody. Antibody detection was performed with a standard avidin-biotin complex detection system after which they were developed with 3,3-diaminobenzidine tetrahydrochloride as the chromogenic substrate (Vectastain avidin-biotin complex, Vector Laboratories, Burlingame, California). Sections were mounted with Pertex (Histolab Products AB, Gothenburg, Sweden) and examined and photographed (Olympus BHS photomicrographic system).

### Solid Phase Binding Assay

Collagen, diluted to 5 µg/mL in 1% acetic acid +1% NaCl, 100 µL/ well, was coated on 96-well flexible assay plates (Falcon 3912, Beckton Dickinson, Oxnard, CA, USA) by incubation at 4°C overnight. The wells were blocked with 1% BSA in PBS +0.1% Tween 20 for 1 hour at RT. Unlabeled A1M in 50 µL PBS +0.05% Tween was added to the wells, followed by 50 µL of the same buffer containing 50×10^3^ cpm ^125^I-labeled A1M and incubated overnight at 4°C. The wells were washed and the activity was determined in a Wallac Wizard 1470 gamma counter.

### Statistical Analysis

Statistical analysis was performed using Origin 8 software (Microcal, Northampton, MA, USA). Students t-test was used for statistical evaluation and was considered significant when *P*<0.05.
